# Supplementary material for: Modeling non-genetic information dynamics in cells using reservoir computing
Source: iScience. 2024 Mar 28;27(4):109614. doi: 10.1016/j.isci.2024.109614 (PMC11022048; doi:10.1016/j.isci.2024.109614)
Supplement: Document S1. Figures S1–S28 [file mmc1.pdf]

iScience, Volume *27*

Supplemental information

Modeling non-genetic information dynamics in cells using reservoir computing

Dipesh Niraula, Issam El Naqa, Jack Adam Tuszynski, and Robert A. Gatenby

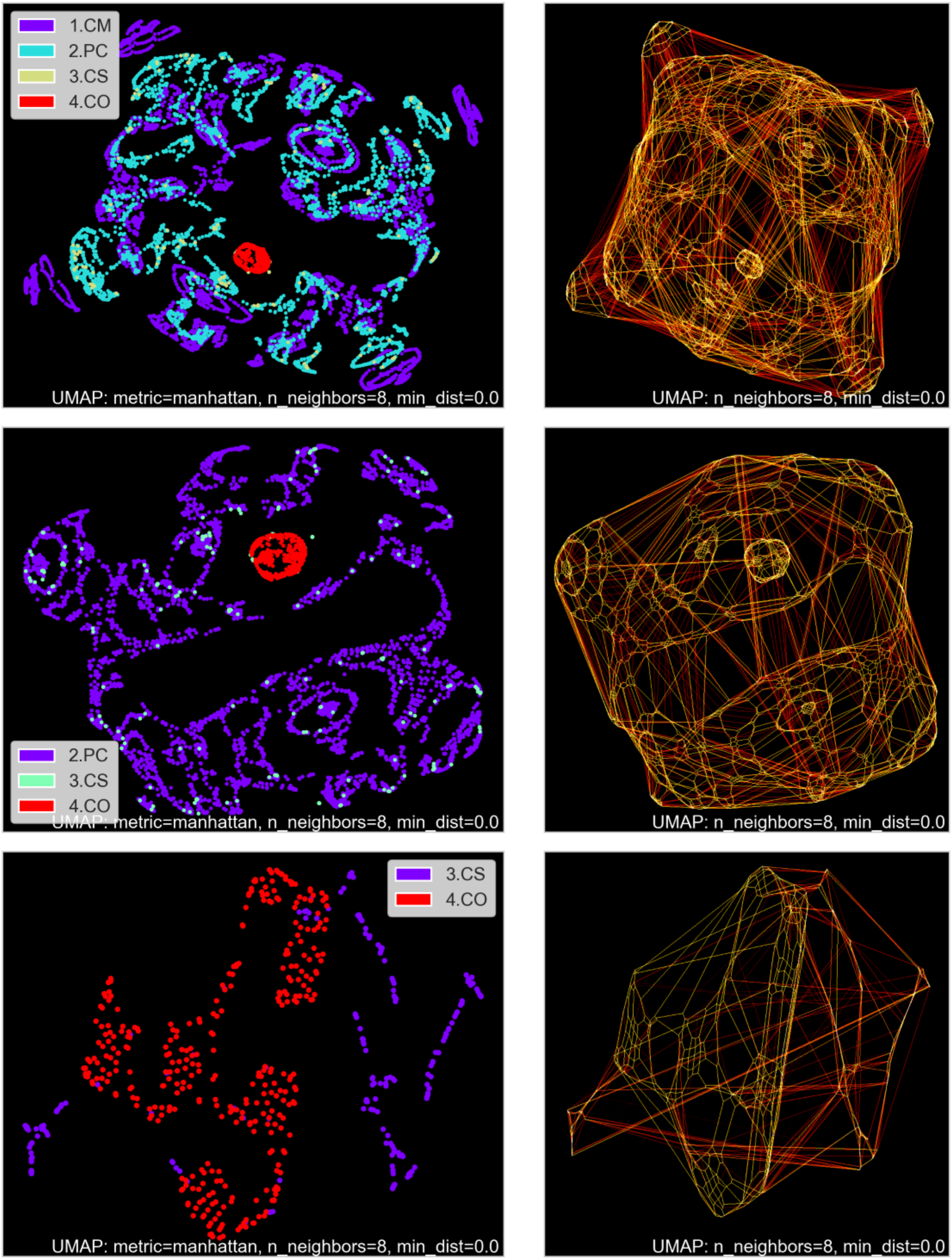

Figure S1: Two dimensional UMAP of three-dimensional Cell-Reservoir for 1% Cytoskeleton Volume, related to Figure 6. The three rows correspond to projections of same cell with all components, without CM, and without CM and PC, respectively. The right column shows UMAP's connection between the nearest neighbor. Abbreviation, CM: cell membrane; PC: peripheral cytoplasm; CS: cytoskeleton; and CO: central organelle.

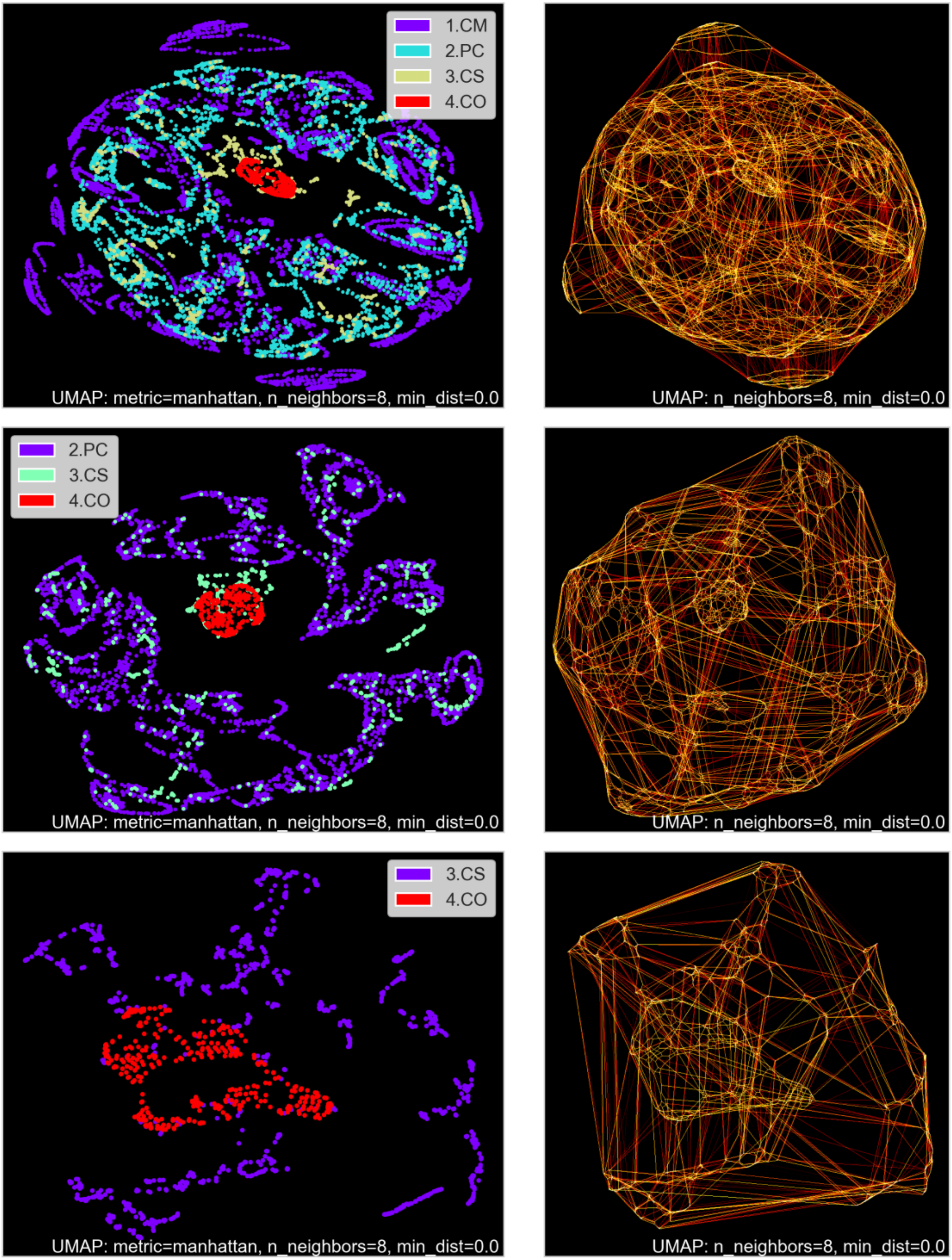

Figure S2: Two dimensional UMAP of three-dimensional Cell-Reservoir for 5% Cytoskeleton Volume, related to Figure 6. The three rows correspond to projections of same cell with all components, without CM, and without CM and PC, respectively. The right column shows UMAP's connection between the nearest neighbor. Abbreviation, CM: cell membrane; PC: peripheral cytoplasm; CS: cytoskeleton; and CO: central organelle.

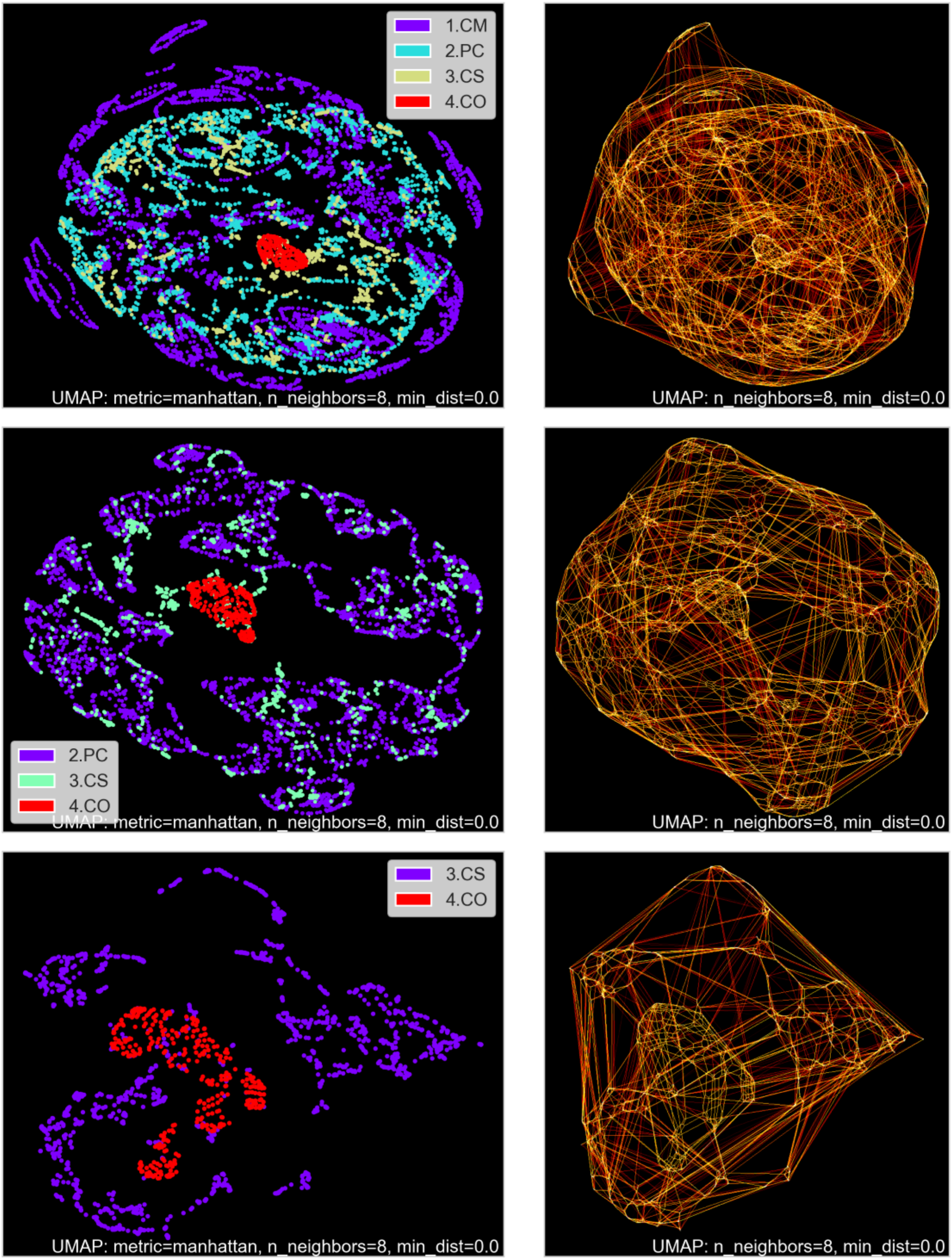

Figure S3: Two dimensional UMAP of three-dimensional Cell-Reservoir for 7% Cytoskeleton Volume, related to Figure 6. The three rows correspond to projections of same cell with all components, without CM, and without CM and PC, respectively. The right column shows UMAP's connection between the nearest neighbor. Abbreviation, CM: cell membrane; PC: peripheral cytoplasm; CS: cytoskeleton; and CO: central organelle.

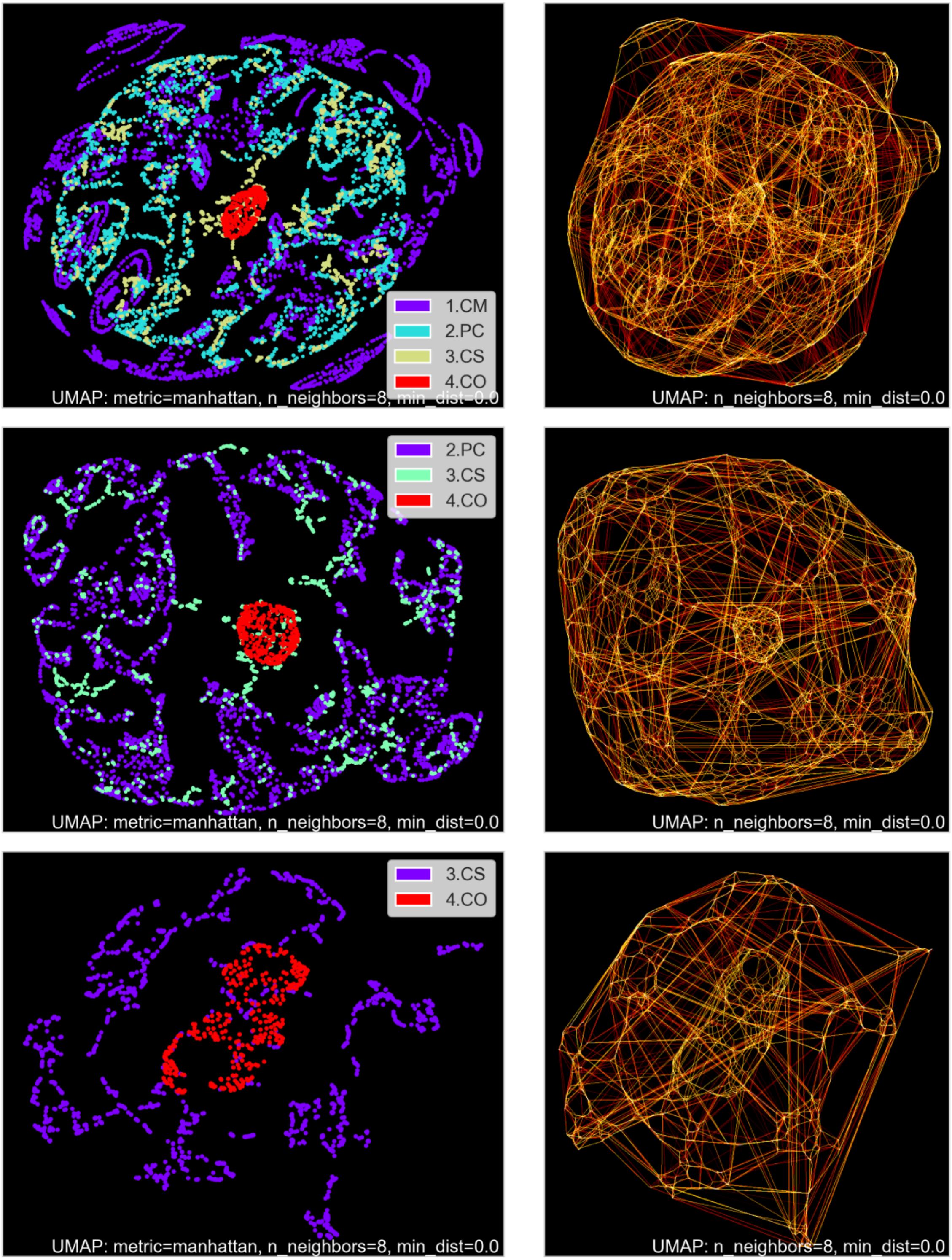

Figure S4: Two dimensional UMAP of 3D Cell-Reservoir for 8% Cytoskeleton Volume, related to Figure 6. The three rows correspond to projections of same cell with all components, without CM, and without CM and PC, respectively. The right column shows UMAP's connection between the nearest neighbor. Abbreviation, CM: cell membrane; PC: peripheral cytoplasm; CS: cytoskeleton; and CO: central organelle.

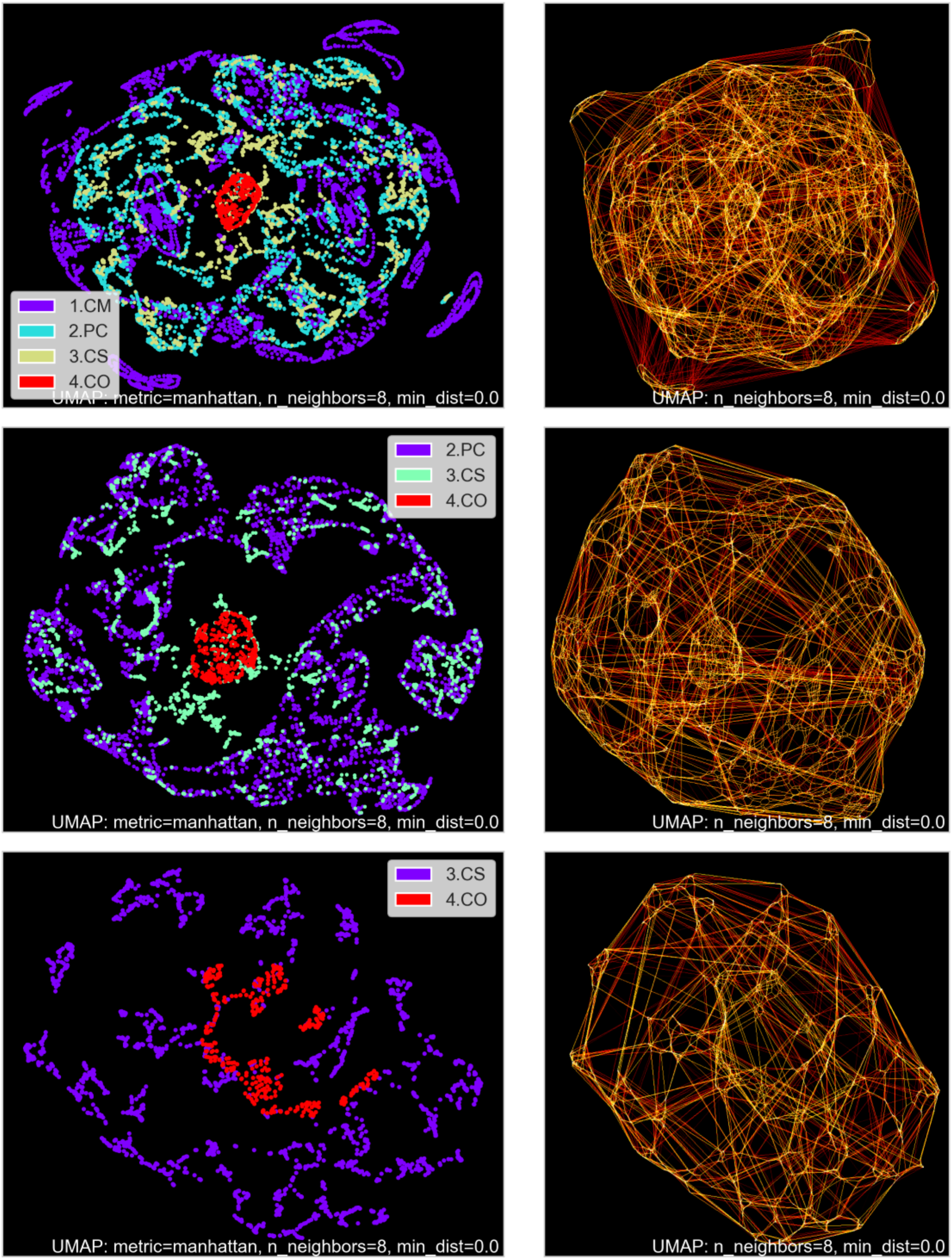

Figure S5: Two dimensional UMAP of 3D Cell-Reservoir 10% Cytoskeleton Volume, related to Figure 6. The three rows correspond to projections of same cell with all components, without CM, and without CM and PC, respectively. The right column shows UMAP's connection between the nearest neighbor. Abbreviation, CM: cell membrane; PC: peripheral cytoplasm; CS: cytoskeleton; and CO: central organelle.

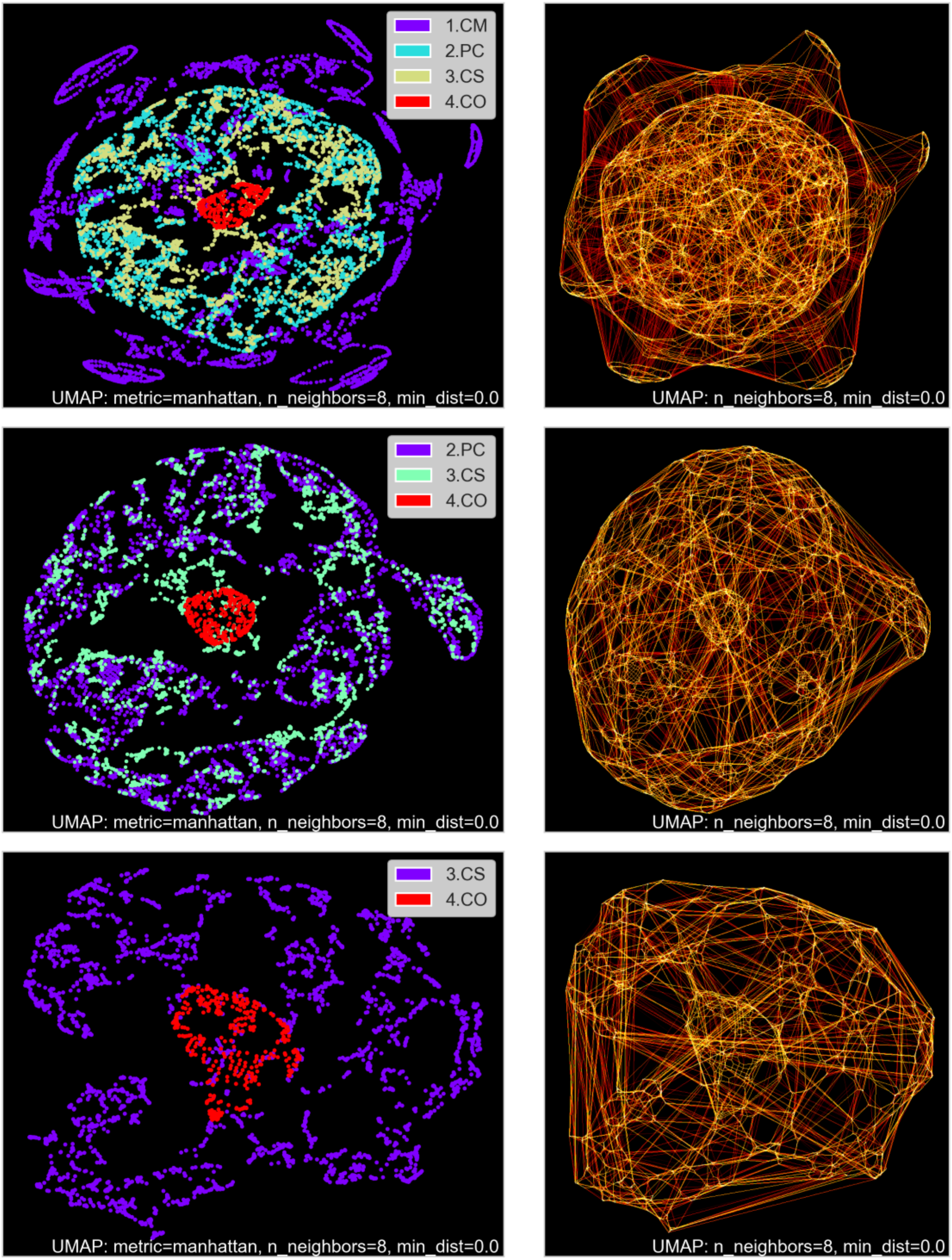

Figure S6: Two dimensional UMAP of 3D Cell-Reservoir for 15% Cytoskeleton Volume, related to Figure 6. The three rows correspond to projections of same cell with all components, without CM, and without CM and PC, respectively. The right column shows UMAP's connection between the nearest neighbor. Abbreviation, CM: cell membrane; PC: peripheral cytoplasm; CS: cytoskeleton; and CO: central organelle.

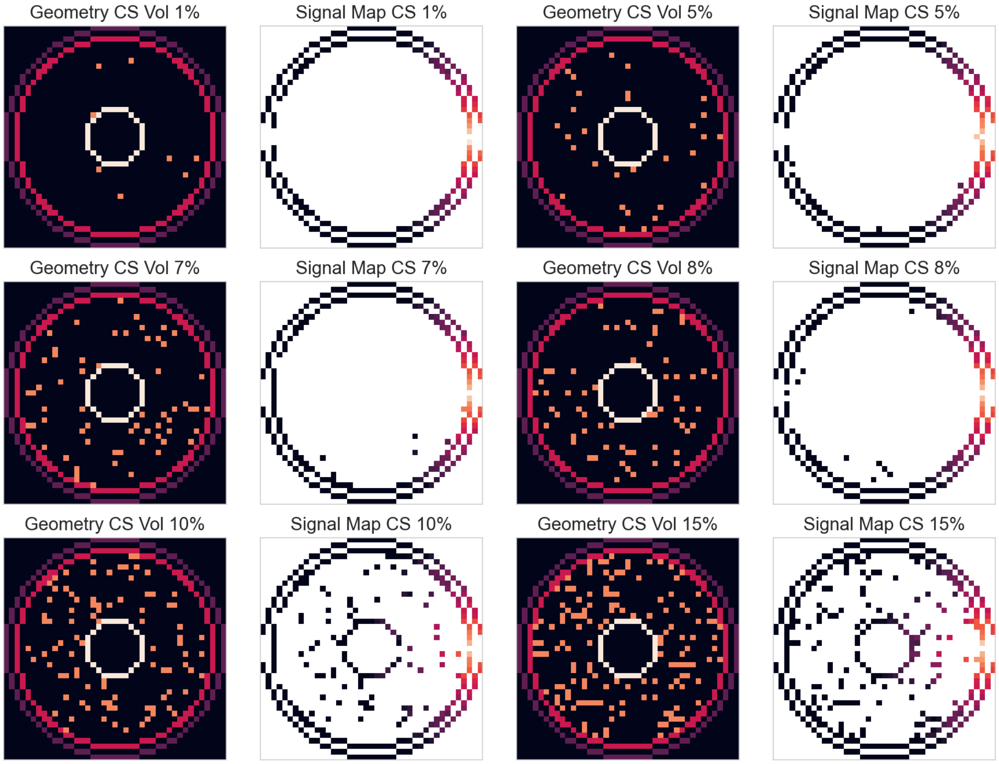

Figure S7: Two dimensional cross-sectional geometry of three-dimensional Cell-Reservoir and corresponding signal map generated by point source for various cytoskeleton volume, related to Figure 6. Abbreviation, CS: cytoskeleton.

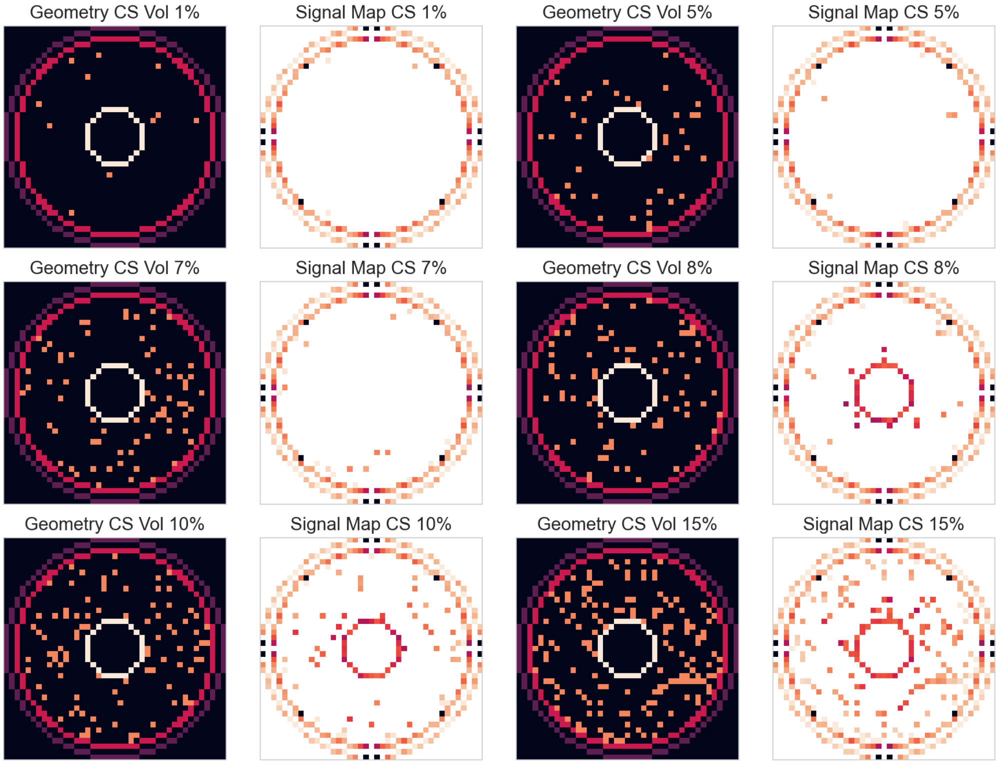

Figure S8: Two dimensional cross-sectional geometry of three-dimensional Cell-Reservoir and corresponding signal distribution generated by spherical source for various cytoskeleton volume, related to Figure 6. Abbreviation, CS: cytoskeleton.

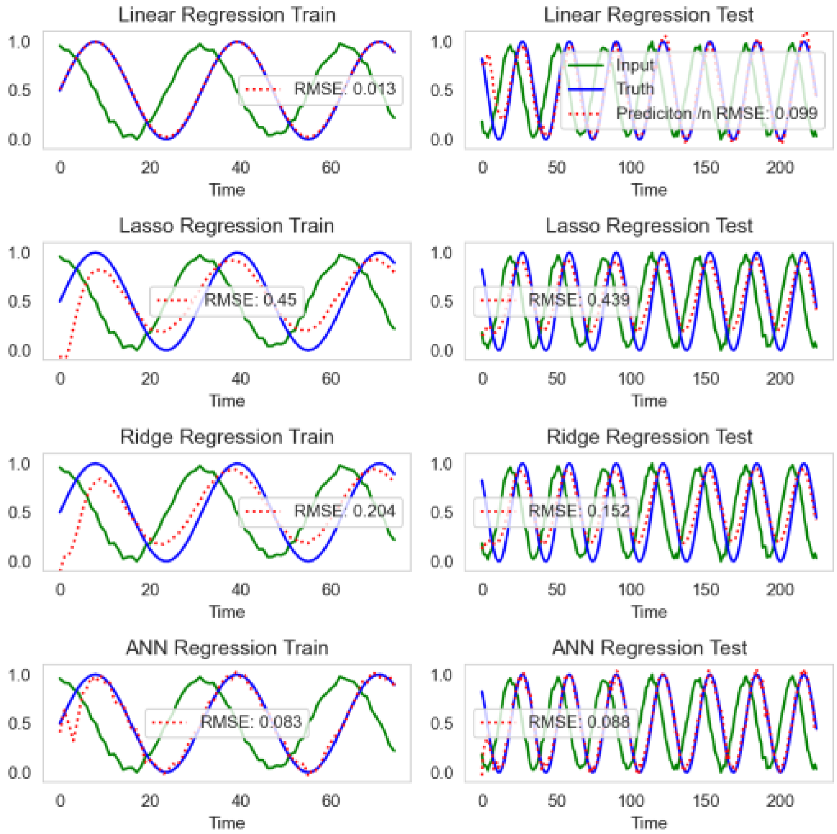

Figure S9: Cell-Reservoir's sine response to noisy cosine perturbation of 0.05 noise-to-signal ratio generated by point source for Linear, Lasso, Ridge, and Artificial Neural Network (ANN) decision-makers, related to Figure 7.

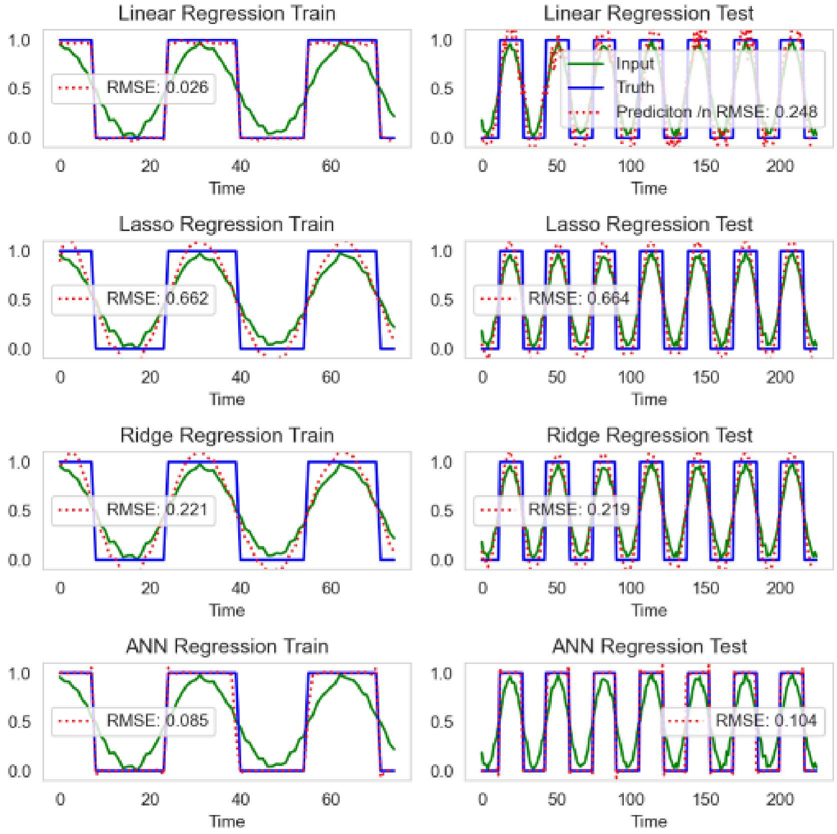

Figure S10: Cell-Reservoir's sine response to noisy step perturbation of 0.05 noise-to-signal ratio generated by point source for Linear, Lasso, Ridge, and Artificial Neural Network (ANN) decision-makers, related to Figure 7.

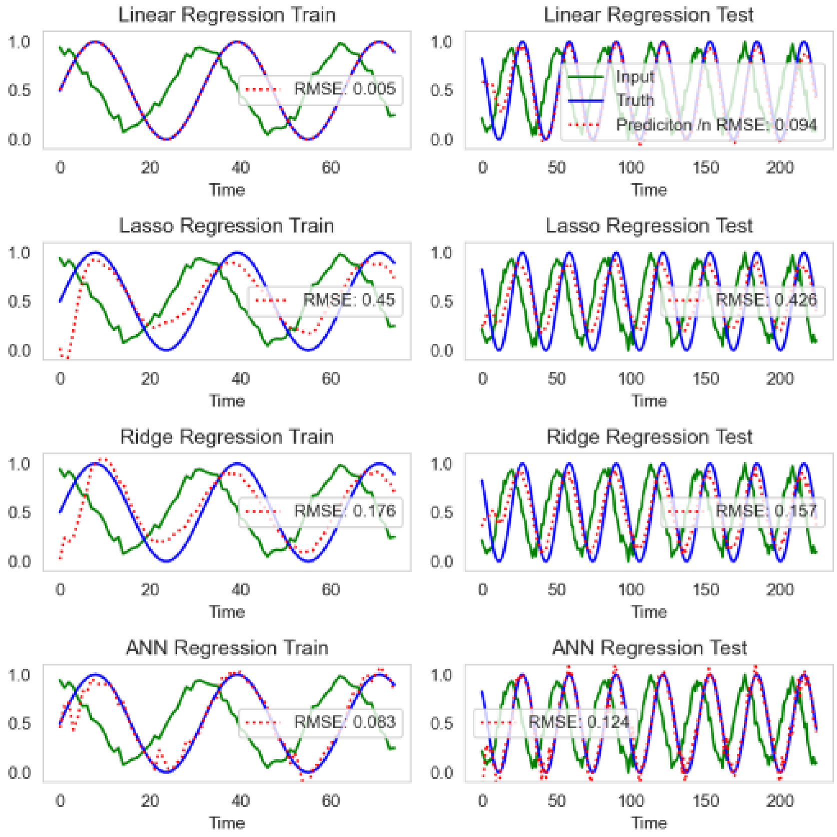

Figure S11: Cell-Reservoir's sine response to noisy cosine perturbation of 0.1 noise-to-signal ratio generated by point source for Linear, Lasso, Ridge, and Artificial Neural Network (ANN) decision-makers, related to Figure 7.

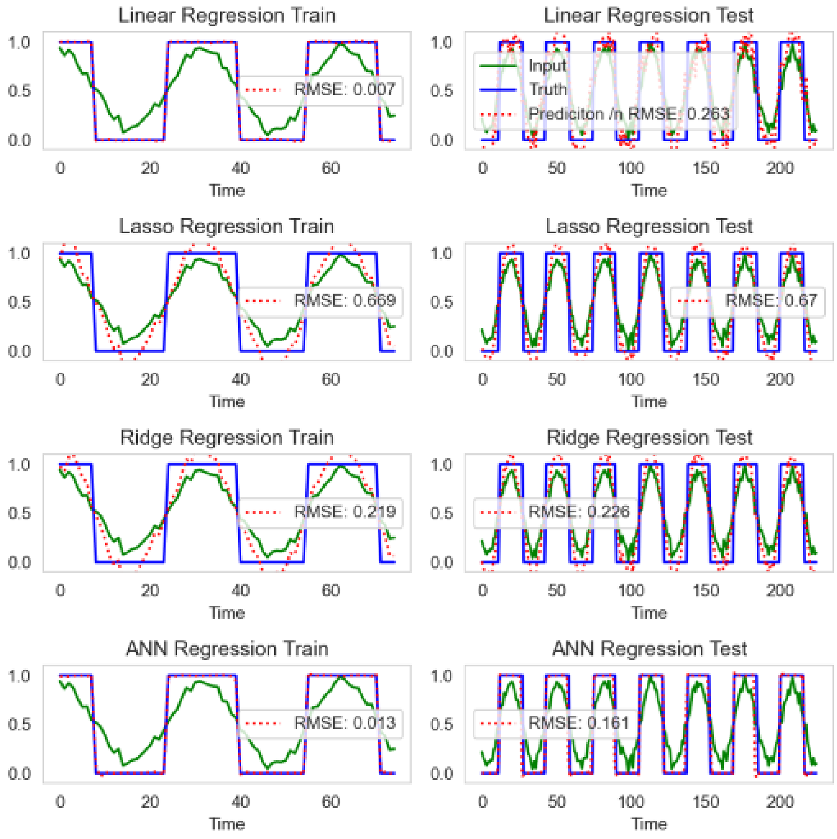

Figure S12: Cell-Reservoir's sine response to noisy step perturbation of 0.1 noise-to-signal ratio generated by point source for Linear, Lasso, Ridge, and Artificial Neural Network (ANN) decision-makers, related to Figure 7.

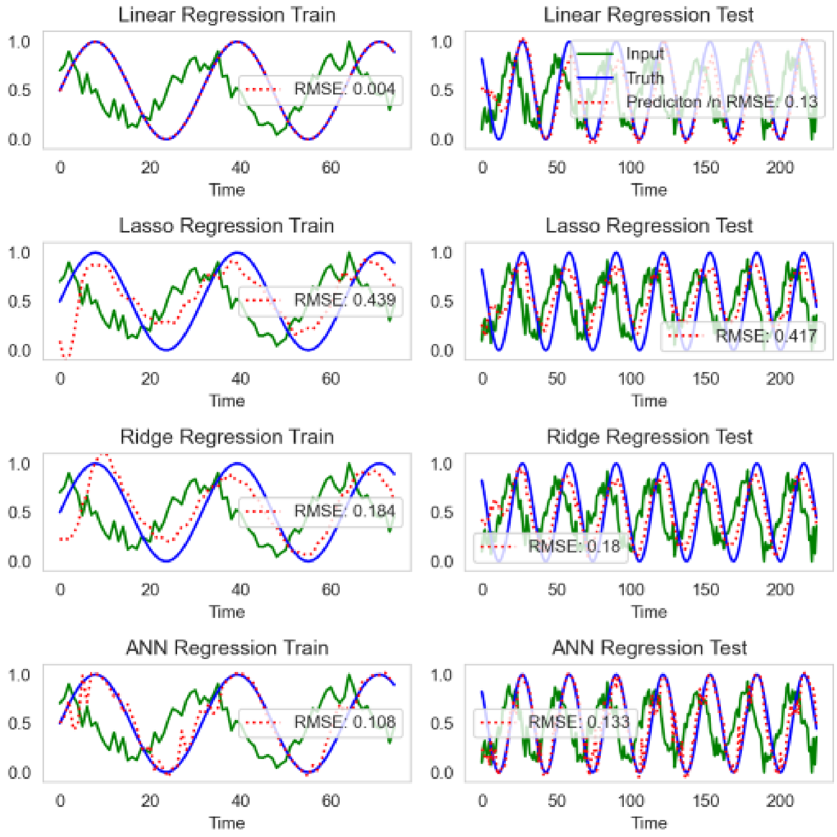

Figure S13: Cell-Reservoir's sine response to noisy cosine perturbation of 0.25 noise-to-signal ratio generated by point source for Linear, Lasso, Ridge, and Artificial Neural Network (ANN) decision-makers, related to Figure 7.

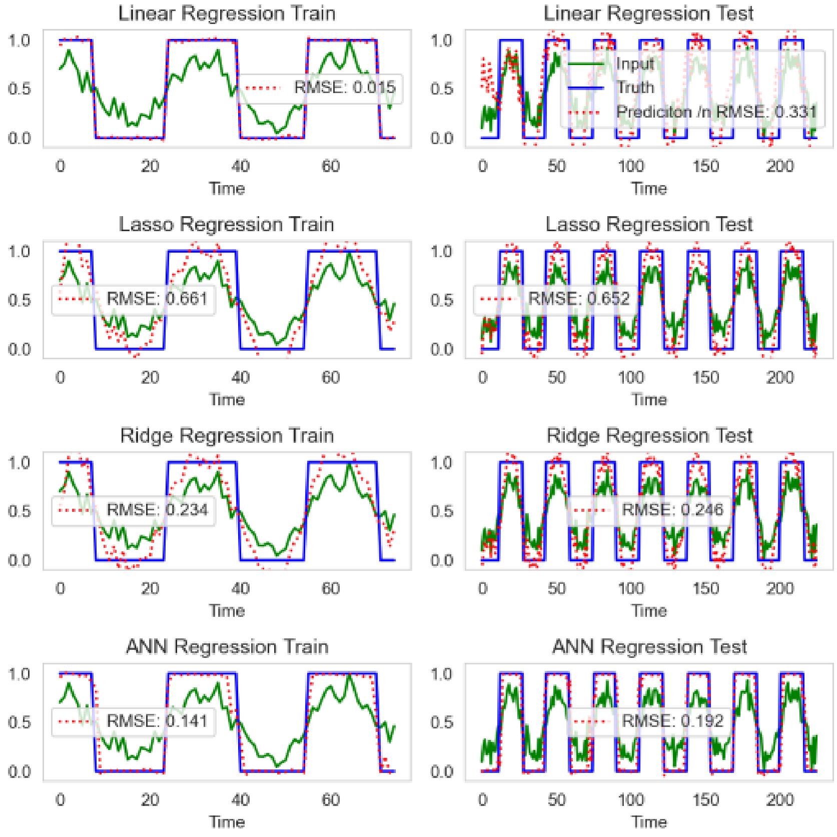

Figure S14: Cell-Reservoir's sine response to noisy step perturbation of 0.25 noise-to-signal ratio generated by point source for Linear, Lasso, Ridge, and Artificial Neural Network (ANN) decision-makers, related to Figure 7.

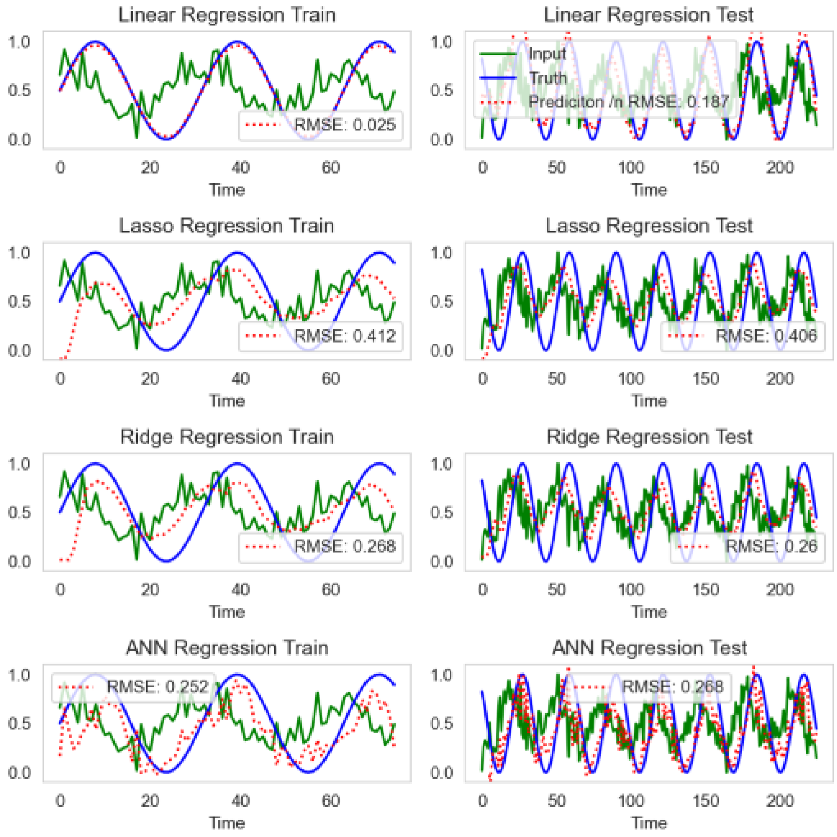

Figure S15: Cell-Reservoir's sine response to noisy cosine perturbation of 0.5 noise-to-signal ratio generated by point source for Linear, Lasso, Ridge, and Artificial Neural Network (ANN) decision-makers, related to Figure 7.

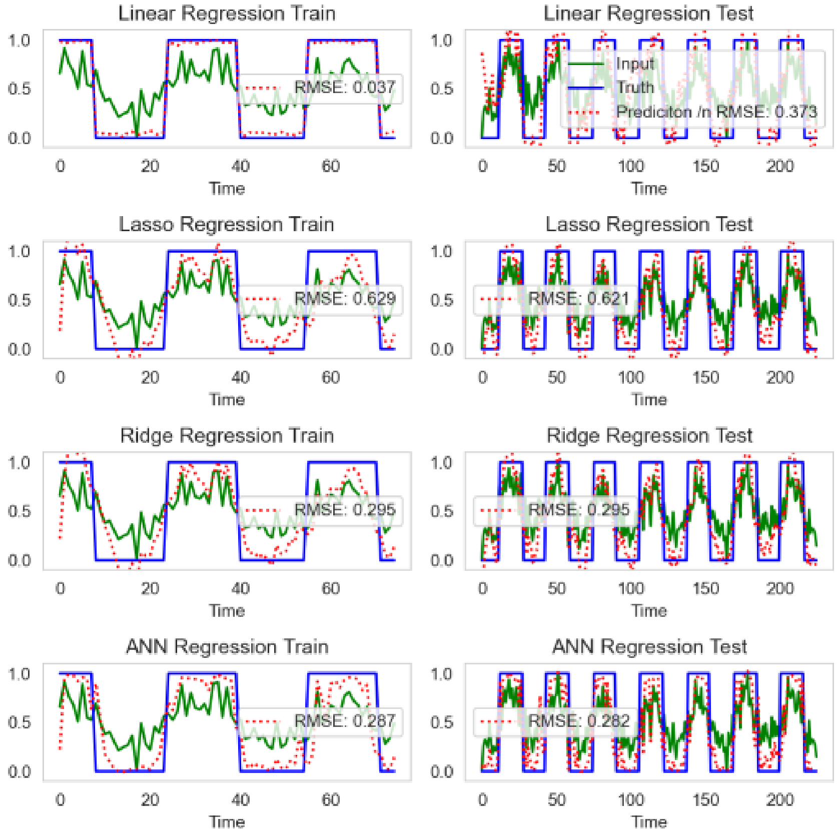

Figure S16: Cell-Reservoir's sine response to noisy step perturbation of 0.5 noise-to-signal ratio generated by point source for Linear, Lasso, Ridge, and Artificial Neural Network (ANN) decision-makers, related to Figure 7.

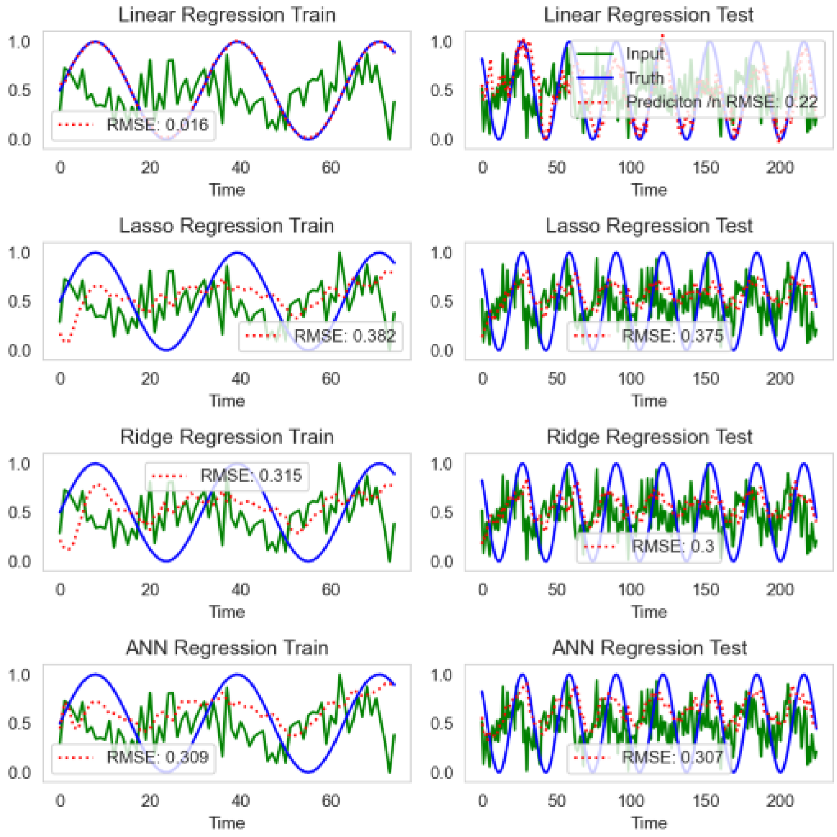

Figure S17: Cell-Reservoir's sine response to noisy cosine perturbation of 1.0 noise-to-signal ratio generated by point source for Linear, Lasso, Ridge, and Artificial Neural Network (ANN) decision-makers, related to Figure 7.

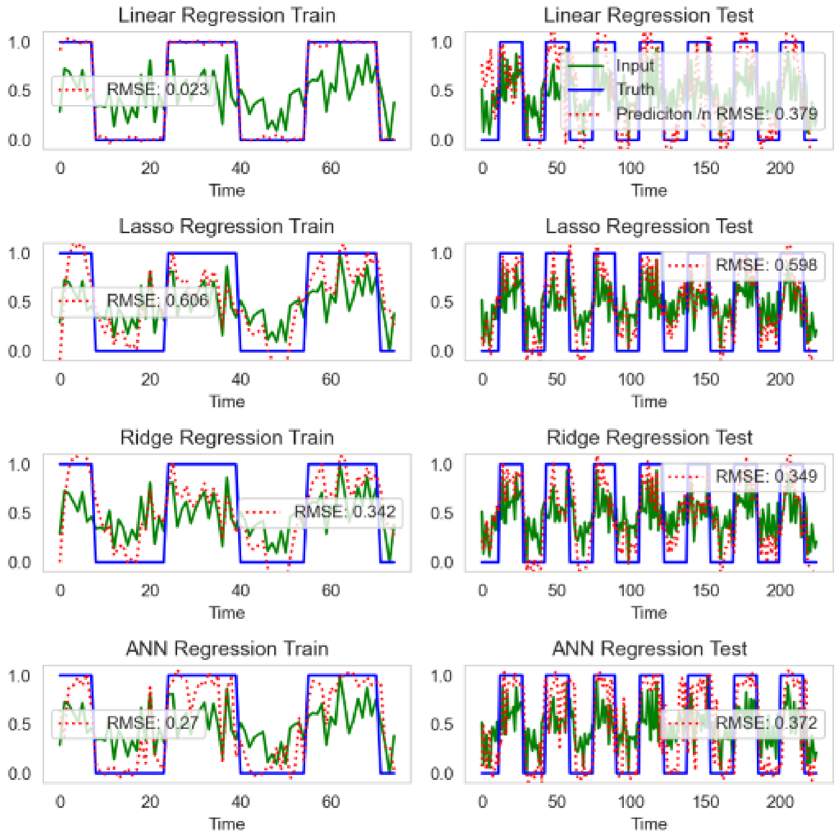

Figure S18: Cell-Reservoir's sine response to noisy step perturbation of 1.0 noise-to-signal ratio generated by point source for Linear, Lasso, Ridge, and Artificial Neural Network (ANN) decision-makers, related to Figure 7.

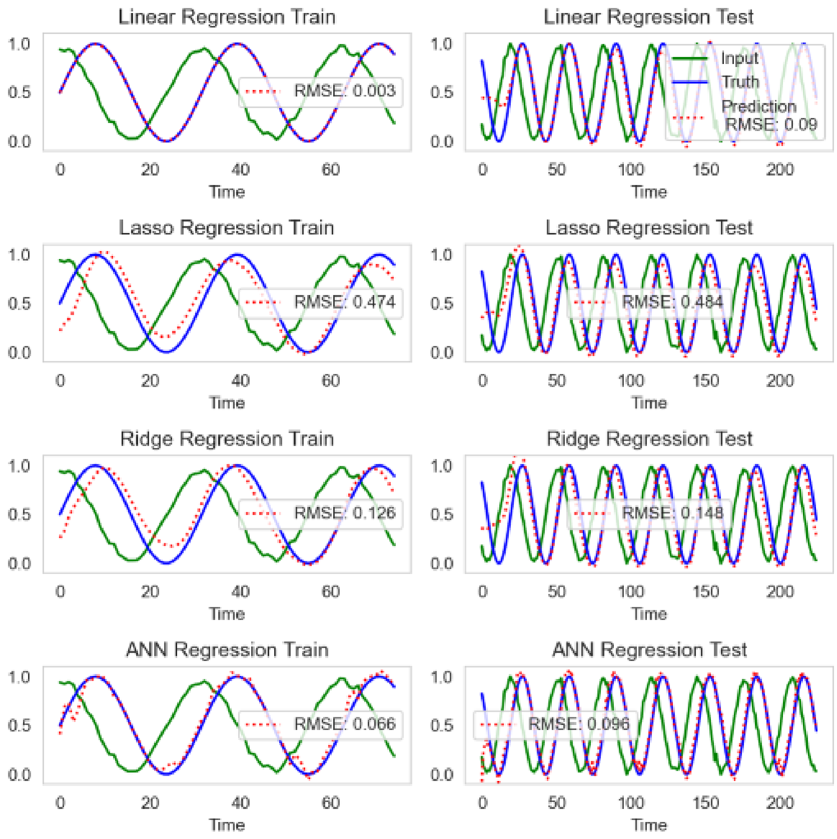

Figure S19: Cell-Reservoir's sine response to noisy cosine perturbation of 0.05 noise-to-signal ratio generated by spherical source for Linear, Lasso, Ridge, and Artificial Neural Network (ANN) decision-makers, related to Figure 7.

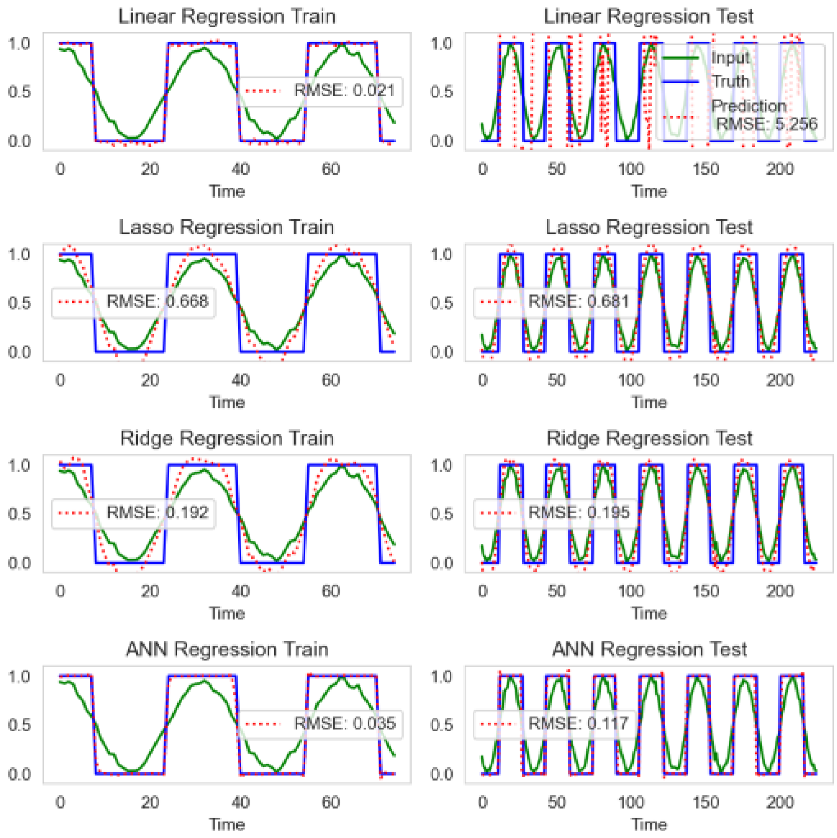

Figure S20: Cell-Reservoir's sine response to noisy step perturbation of 0.05 noise-to-signal ratio generated by spherical source for Linear, Lasso, Ridge, and Artificial Neural Network (ANN) decision-makers, related to Figure 7.

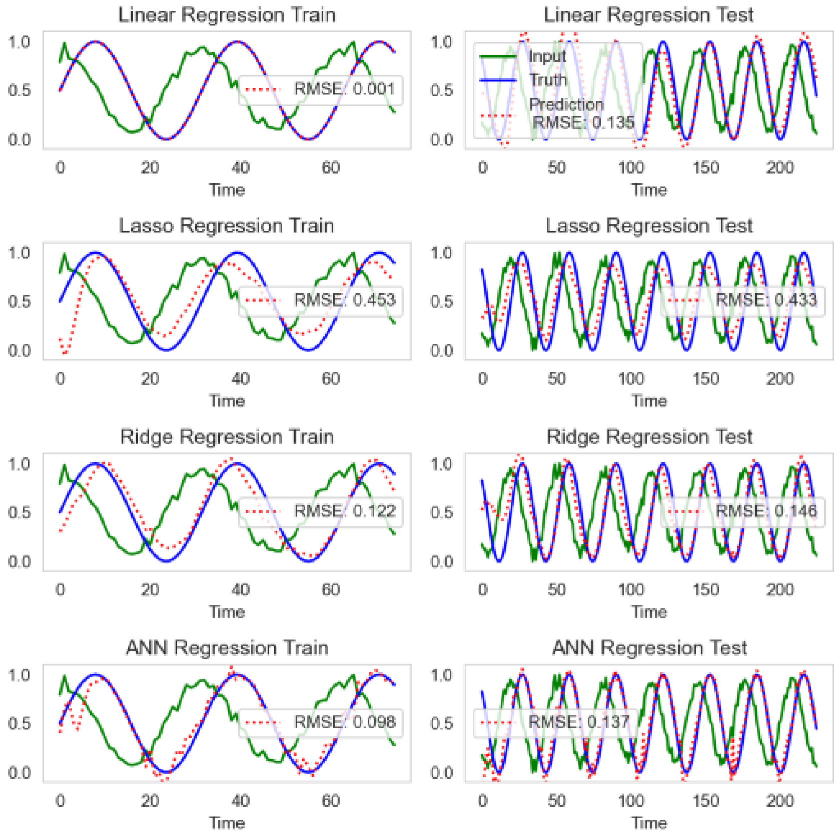

Figure S21: Cell-Reservoir's sine response to noisy cosine perturbation of 0.1 noise-to-signal ratio generated by spherical source for Linear, Lasso, Ridge, and Artificial Neural Network (ANN) decision-makers, related to Figure 7.

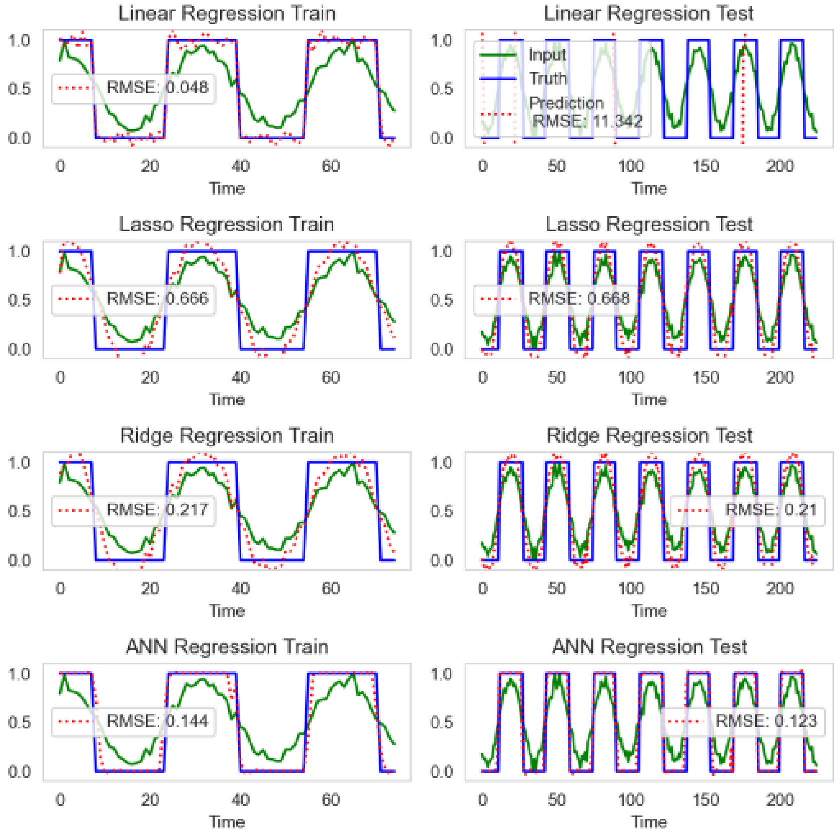

Figure S22: Cell-Reservoir's sine response to noisy step perturbation of 0.1 noise-to-signal ratio generated by spherical source for Linear, Lasso, Ridge, and Artificial Neural Network (ANN) decision-makers, related to Figure 7.

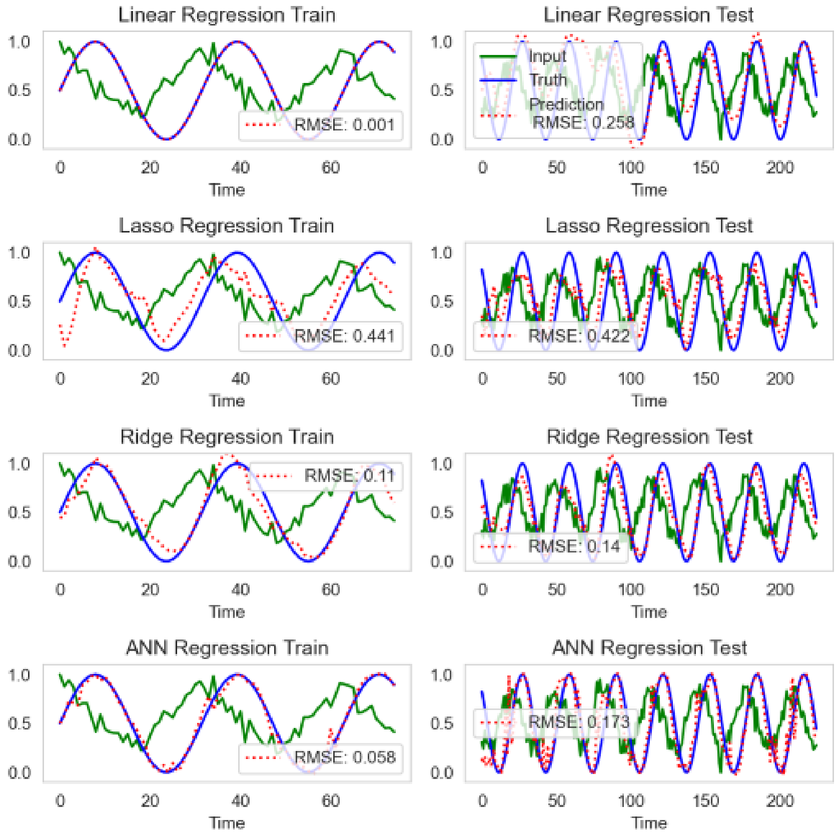

Figure S23: Cell-Reservoir's sine response to noisy cosine perturbation of 0.25 noise-to-signal ratio generated by spherical source for Linear, Lasso, Ridge, and Artificial Neural Network (ANN) decision-makers, related to Figure 7.

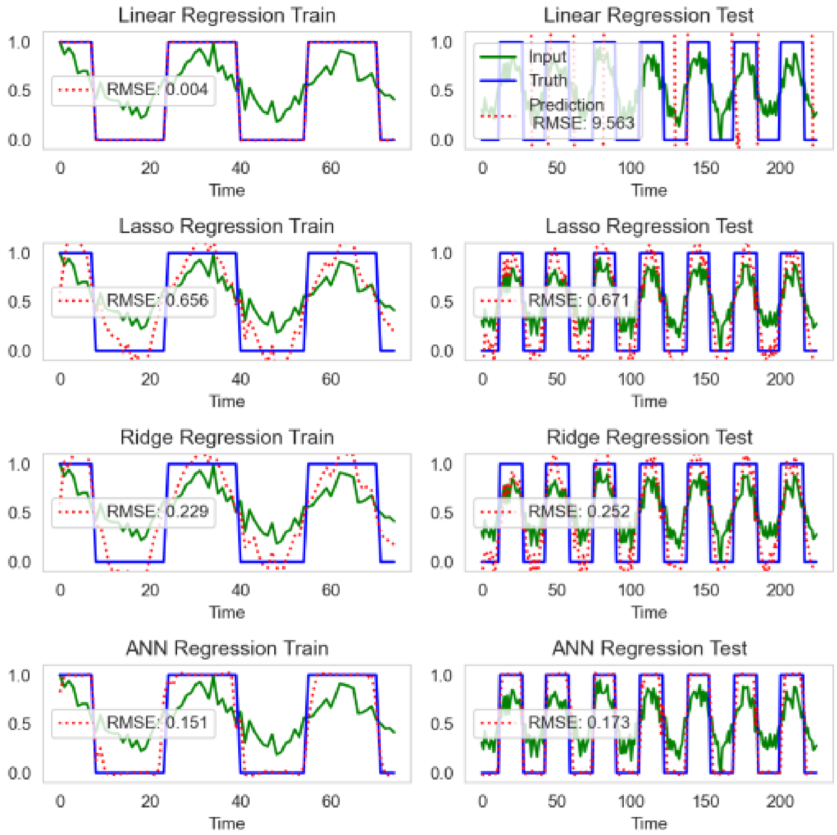

Figure S24: Cell-Reservoir's sine response to noisy step perturbation of 0.25 noise-to-signal ratio generated by spherical source for Linear, Lasso, Ridge, and Artificial Neural Network (ANN) decision-makers, related to Figure 7.

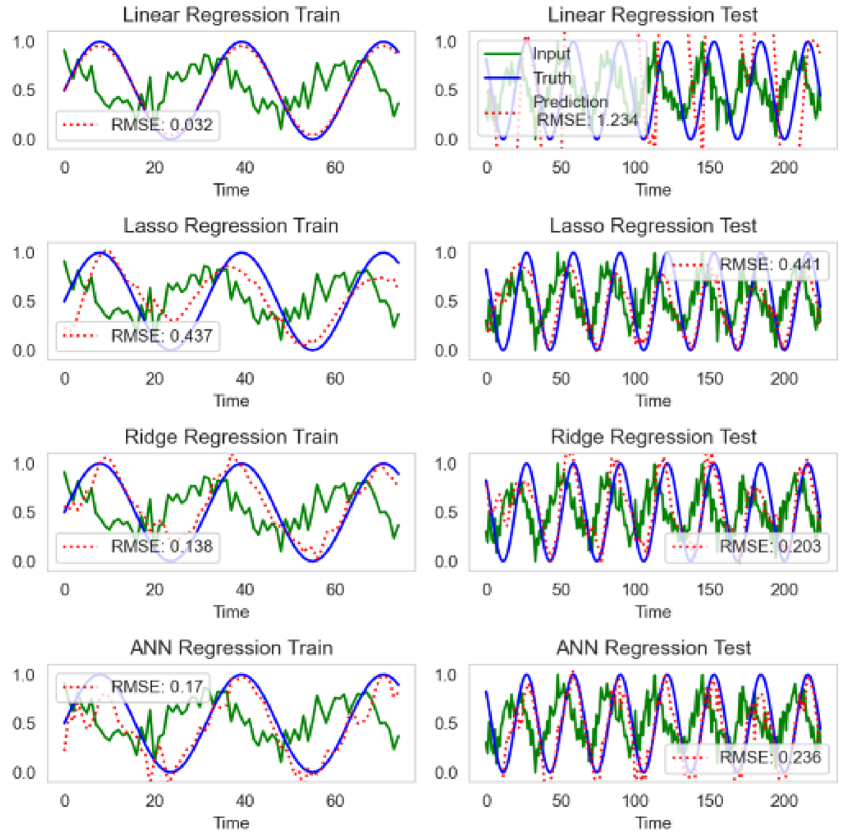

Figure S25: Cell-Reservoir's sine response to noisy cosine perturbation of 0.5 noise-to-signal ratio generated by spherical source for Linear, Lasso, Ridge, and Artificial Neural Network (ANN) decision-makers, related to Figure 7.

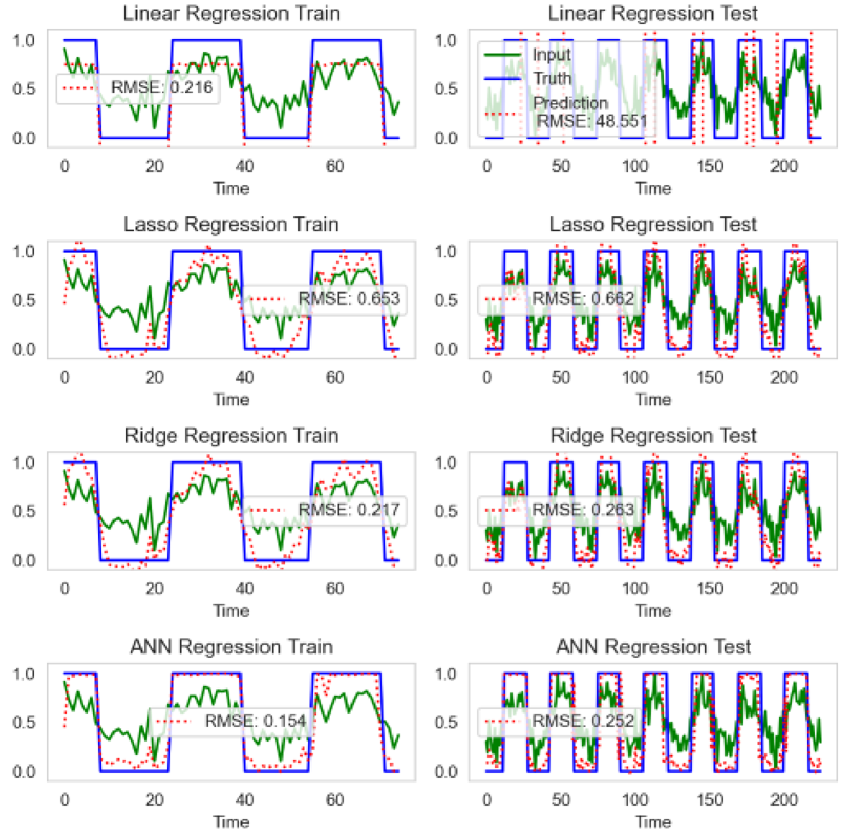

Figure S26: Cell-Reservoir's sine response to noisy step perturbation of 0.5 noise-to-signal ratio generated by spherical source for Linear, Lasso, Ridge, and Artificial Neural Network (ANN) decision-makers, related to Figure 7.

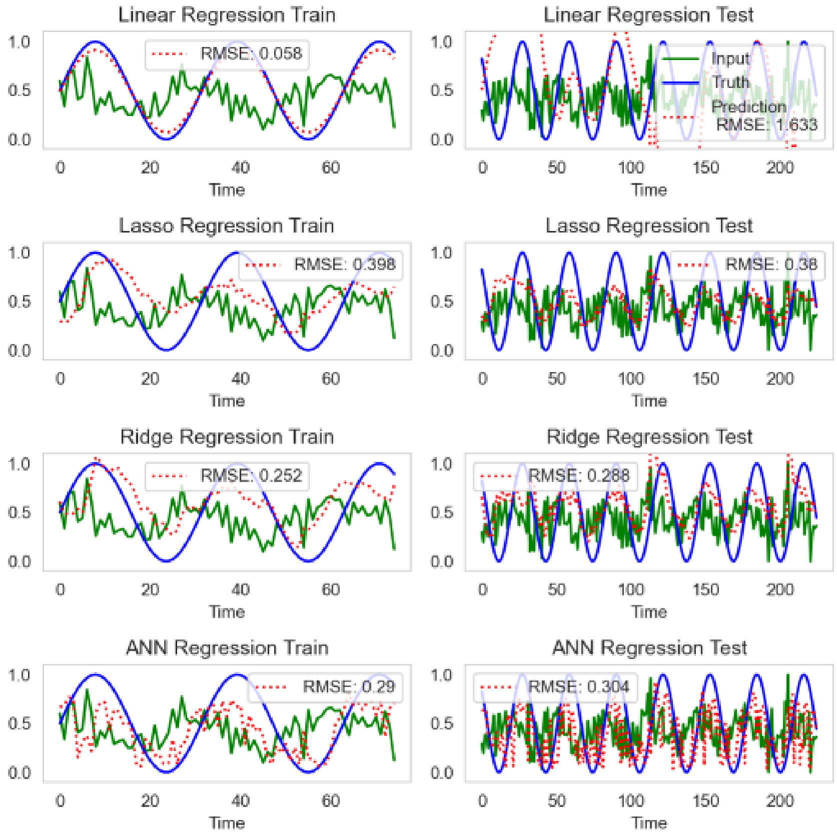

Figure S27: Cell-Reservoir's sine response to noisy cosine perturbation of 1.0 noise-to-signal ratio generated by spherical source for Linear, Lasso, Ridge, and Artificial Neural Network (ANN) decision-makers, related to Figure 7.

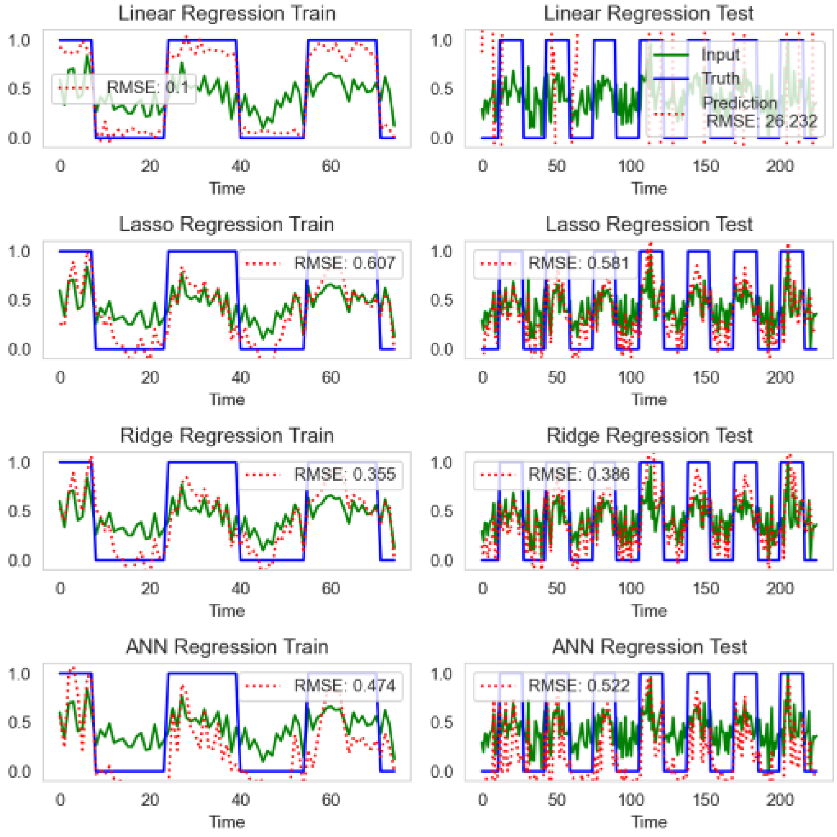

Figure S28: Cell-Reservoir's sine response to noisy step perturbation of 1.0 noise-to-signal ratio generated by spherical source for Linear, Lasso, Ridge, and Artificial Neural Network (ANN) decision-makers, related to Figure 7.
